# Development of Secure Infrastructure for Advancing Generative AI Research in Healthcare at an Academic Medical Center

**DOI:** 10.21203/rs.3.rs-5095287/v1

**Published:** 2024-09-24

**Authors:** Madelena Y. Ng, Jarrod Helzer, Michael A. Pfeffer, Tina Seto, Tina Hernandez-Boussard

**Affiliations:** Stanford University; Stanford Health Care; Stanford University; Stanford Health Care; Stanford University

## Abstract

The increasing interest in leveraging generative AI models in healthcare necessitates secure infrastructure at academic medical centers. Without an all-encompassing secure system, researchers may create their own insecure microprocesses, risking the exposure of protected health information (PHI) to the public internet or its inadvertent incorporation into AI model training. To address these challenges, our institution implemented a secure pathway to the Azure OpenAI Service using our own private OpenAI instance which we fully control to facilitate high-throughput, secure LLM queries. This pathway ensures data privacy while allowing researchers to harness the capabilities of LLMs for diverse healthcare applications. Our approach supports compliant, efficient, and innovative AI research in healthcare. This paper discusses the implementation, advantages, and use cases of this secure infrastructure, underscoring the critical need for centralized, secure AI solutions in academic medical environments.

## Background

The use of generative artificial intelligence (AI) tools such as large language models (LLMs) in healthcare has the potential to improve patient care, alleviate clinical burden, and enhance operational efficiency. Despite these upsides, leveraging LLMs in healthcare is complicated due to complex regulations, including data security and patient privacy.^1^ Researchers often fine-tune existing pre-trained LLMs (e.g., GPT, LLaMA, and PaLM) with clinical data, but these adjusted models become more susceptible to security threats and privacy leakage attacks.^2^ Furthermore, data pre-processing decisions vary drastically across research teams, which can lead to integrating sensitive or protected health information (PHI) into LLMs, creating security, privacy, and regulatory risks.^3^ Thus, academic medical centers^4,5^ are investing in secure infrastructure to train the next generation of researchers and learners, thereby enabling clinical LLM experimentation to scale safely and meet the pace of innovation.

In January 2024, our healthcare institution launched “Secure GPT” chat web application for our research community to query GPT 4.0 with PHI and/or PII in a closed system. The Secure GPT web app, similar to ChatGPT, operates in a secured cloud environment to ensure no data passed through is retained or modifies the model. However, its manual single prompt approach did not meet researcher or learner needs. To address this, we developed a secure pathway to the OpenAI application programming interface (API), to enable our community to seamlessly access and integrate Secure GPT’s capabilities to create diverse, multi-modal healthcare use cases. To further safeguard this gateway from internal and external risks, we describe the security measures undertaken to ensure a secure environment for generative AI research, aiming to share our work and foster innovation in healthcare and beyond.

## Methods

Stanford Medicine’s information technology department, Technology and Digital Solutions (TDS), implemented a secure infrastructure for medical researchers, clinicians and in-house application developers to have private API-enabled endpoints to a dedicated Azure OpenAI Studio deployment. The models available in the deployment (at the time of writing) include GPT-4o, GPT-4, GPT-4–32k, GPT-3.5-Turbo, text-embedding ADA-002 and DALL-E 3. The infrastructure team can deploy models on demand for each instance.

To establish the secure infrastructure, TDS created a dedicated Azure subscription for hosting OpenAI Studio, supported by an existing business associate agreement (BAA) with Microsoft Azure and Stanford Health Care. This subscription can hold up to 30 OpenAI instances per region, with various tokens per minute (TPM) limits depending on the LLM used and limits granted. For example, a total of 10 million TPM/60,000 requests per minute could be allowed for the GPT-4o model in a subscription. Each instance has an initial call rate limit of 3,000 TPM, adjustable upon request. As proof of concept, TDS configured an OpenAI instance for one research lab, providing a private URL endpoint and a secure API key stored in HashiCorp’s Vault (https://vault.med.stanford.edu) to ensure privacy and security.

Two Azure ExpressRoute circuits establish connectivity that securely extend the on-premise network into Microsoft cloud services via a private connection, ensuring data does not travel via the public internet. This setup not only enhances security, but it also provides fast and reliable connection with consistent low latency. Private IP addresses and their corresponding names are registered in the School of Medicine’s directory. This allows Stanford Health Care’s Azure endpoints to be accessed securely from the School of Medicine and through both organizations’ private networks. With the private endpoint IP address hosted on Stanford’s network, any data submitted to the models stays within a secure and private environment. This ensures that sensitive information remains protected and accessible only within the approved network.

This secure infrastructure was developed through collaboration across the institution, including cloud engineering, security, and networking teams. By providing a secure, centrally managed environment, individual research labs avoid building their own microprocesses, which may have their own inherent risk. Costs can be recovered based on usage, with labs able to monitor their usage and costs via a dashboard.

To complete the secure infrastructure, researchers utilize a private GitLab (https://gitlab.med.stanford.edu/som-labs) for code source control and versioning, rather than the public GitHub. All software and systems within this infrastructure are managed and maintained by the cloud engineering team. By integrating all components into the TDS toolchain, a comprehensive, secure solution for researchers, clinicians and staff to access generative AI models efficiently and safely could be obtained.

## Use Case

To demonstrate the system’s effectiveness, we provide two use cases that involved securely accessing and processing patient data through Stanford Health Care’s Azure endpoints. Institutional Review Board (IRB) approval was obtained to ensure compliance with ethical standards and protect patient privacy.

### Use Case: Fall Detection Using Secure GPT API

To improve fall detection capabilities in healthcare settings, our team leveraged Stanford’s private API for Secure GPT to assess the effectiveness of LLMs, including GPT-4, in detecting post-operative fall events from EHR clinical notes. This use case focused on processing clinical notes from a cohort of surgical patients, extracting sentences mentioning falls and their context using regex protocols. By conducting zero-shot and few-shot prompting, we evaluated the model’s performance on a random sample of notes. The models demonstrated superior capabilities in detecting falls compared to traditional NLP methods, which have shown only mediocre discrimination capabilities. This approach exemplifies how the API can process clinical notes effectively, ensuring data privacy while leveraging advanced LLM capabilities to enhance clinical outcomes. Our findings were further validated in an external healthcare setting, highlighting the potential for scalable and secure AI integration in clinical practice.^6^

### Use Case: Evaluating Bias in Mental Health Analysis with Secure GPT API

Utilizing the Secure GPT API, we assessed the performance and bias of different LLMs across eight mental health conditions using clinical notes obtained from the EHR. We integrated ten LLMs of varying sizes, including TinyLlama-1.1B, Gemma-2B, Phi-3-mini, Gemma-7b, Llama-2–7b, Llama-2–13b, MentaLLaMA-7B, MentaLLaMA-13B, Llama-3–8B, and GPT-4. We evaluated each model’s performance and fairness in tasks such as depression and stress prediction. We employed fairness-aware prompts to reduce biases and analyzed results for demographic disparities. Domain-specific models outperformed larger LLMs, with GPT-4 achieving the best balance between performance and fairness. Fairness-aware prompts were effective in reducing bias without significantly compromising performance. The secure infrastructure and API facilitated a comprehensive evaluation of biases in LLMs, advancing equitable AI research and underscoring the importance of targeted prompting strategies and secure data handling.^7^

## Discussion

Generative AI models, especially LLMs, are increasingly being integrated into healthcare systems, presenting both opportunities and challenges. While LLMs may improve patient care, reduce clinical burdens, and enhance operational efficiency, their use with PHI raises significant security and privacy concerns. Thus, academic medical centers will inevitably be confronted with the need to modernize their infrastructure for leveraging LLMs safely within a controlled environment. It becomes untenable to expect researchers and learners to individually figure out appropriate processes. Through our development of a secure gateway to OpenAI’s API, we prevent the creation of ad-hoc, unsecured microprocesses, and systematically safeguard sensitive health information and ensure compliance with regulatory standards. Our secure platform not only facilitates high-throughput LLM queries but also promotes innovation and equitable AI research by providing a reliable, private network for clinical data analysis from diverse stakeholders across the organization. Furthermore, we demonstrate returns on generative AI research quality and precision, which extends the bounds of what constitutes best practices for Responsible AI. By sharing our work, we aim to provide a blueprint that informs how other academic medical centers can develop secure infrastructure for LLM experimentation and connect with a plethora of promising generative models for clinical innovation.
